# Epidemiological, clinical characteristics and prognostic factors analysis of adult patients with hemophagocytic lymphohistiocytosis in a Chinese hospital

**DOI:** 10.3389/fimmu.2025.1684308

**Published:** 2025-12-12

**Authors:** Mingjun Xie, Yaman Wang, Min Wang, Jun Zhou, Hua-Guo Xu

**Affiliations:** 1 Department of Laboratory Medicine, the First Affiliated Hospital with Nanjing Medical University, Nanjing, Jiangsu, China; 2 Branch of National Clinical Research Center for Laboratory Medicine, Nanjing, Jiangsu, China

**Keywords:** adult HLH, HLH epidemiological, HLH prognosis, HLH treatment, retrospective cohort study

## Abstract

**Background:**

Hemophagocytic lymphohistiocytosis (HLH) is a rare and life-threatening syndrome characterized by immune dysregulation and excessive inflammation. Although diagnostic criteria and treatment protocols of HLH are well-established for pediatric populations, managing adult HLH remains challenging.

**Methods:**

We conducted a single-center retrospective cohort study with adult HLH using data from the First Affiliated Hospital with Nanjing Medical University (January 2015–November 2023). Patient demographics, triggers, and outcomes were analyzed. Trends in case volume, diagnostics, treatments, and 30-day mortality were assessed using Sen’s slope estimator. To evaluate the COVID-19 pandemic’s impact, we compared pre-/post-January 2020 data. Logistic regression, Kaplan-Meier survival analysis and resource utilization analysis were applied in the analysis.

**Results:**

Among 711 HLH patients (71.1% aged 43–78 years), malignancy (45.9%) and infection (31.3%) were the predominant triggers. Cases showed a non-significant upward trend (peak increase: 103.6%; slope=2.458; *p* = 0.348), while 30-day mortality showed a non-significant downward trend (slope=-0.819; *p* = 0.402). Post-pandemic, infectious indicators (e.g., WBC) differed significantly (*p*<0.05), though trigger distribution was unchanged (*p* = 0.790). Malignancy-related HLH who received HLH-specific therapy was associated with a higher survival rate (77.7% vs. 34.1%–63.4%; *p*<0.001). A positive correlation between systemic corticosteroid administration and favorable clinical outcome in geriatric patient cohorts. (≥69 years; 70.7% -75.5% vs. 29.6%–42.9%; *p*<0.001). Mean length of hospital stay (LOS) was 21.4 ± 19.2 days.

**Conclusion:**

Despite advancements in pediatric HLH, adult HLH mortality remains high, driven by diagnostic delays, comorbid complexity, and lack of standardized protocols. Future efforts must prioritize: (1) adult-specific biomarkers for early diagnosis, (2) trigger-tailored immunotherapies, and (3) multidisciplinary care pathways to address multisystem involvement.

## Introduction

Hemophagocytic lymphohistiocytosis (HLH) is a life-threatening syndrome of immune dysregulation, marked by uncontrolled activation of macrophages and T-cells, hypercytokinemia, and subsequent multi-organ damage (1). Its pathogenesis involves diverse triggers (e.g., infections, malignancies, autoimmune disorders) that disrupt immune homeostasis, leading to systemic inflammation. Clinically, HLH presents with non-specific but hallmark features: prolonged fever, cytopenias, hepatosplenomegaly, elevated ferritin (>500 μg/L), and hemophagocytosis on bone marrow biopsy (2).

HLH is classified into primary (familial) and secondary (acquired) forms. Primary HLH, caused by genetic mutations, primarily affects infants and children, with an incidence of ~1.2/100,000 and early mortality rates of 20–30% (3–6). In contrast, secondary HLH—more common in adults—lacks robust epidemiological data but shows higher prevalence in elderly populations and early mortality rates of 30–40%, varying by etiology (7–9).

Recent studies have begun addressing these gaps. A UK cohort (2003–2018) mapped HLH incidence trends (10), while meta-analyses pooled small cohorts to outline triggers/outcomes (11). US studies characterized HLH using clinical/public health data (2), yet no large-scale Chinese cohort has systematically analyzed adult HLH’s clinical spectrum as we known.

To bridge this gap, we undertook a large-scale retrospective cohort study (2015–2023) at the First Affiliated Hospital with Nanjing Medical University—a leading national tertiary referral center. This study was designed to comprehensively assess evolving epidemiological patterns, delineate clinical profiles, identify critical prognostic determinants, and examine disparities in treatment approaches. These generate evidence-based insights that can guide strategic clinical planning and policy-making.

## Methods

### Data sample

We conducted a single-center retrospective cohort study using data from the First Affiliated Hospital with Nanjing Medical University (January 1, 2015–November 1, 2023). Adult inpatients (≥18 years) meeting the HLH-2004 diagnostic criteria were identified. Patients with extremely incomplete data were excluded.

For eligible patients, we systematically extracted baseline characteristics (demographics, comorbidities, complications), etiology (infectious diseases, malignancies, Autoimmune disease group, and Other (including pregnancy, drugs, transplantation, etc.)/No identified underlying cause group.), laboratory parameters, treatment and 30-day mortality, resource utilization. Above data were retrieved from electronic medical records and supplemented by structured telephone follow-up, supplemented by physician/family verification.

HLH-2004 diagnostic criteria were used as reference: HLH can be diagnosed if either of the following two criteria is met: (1) Molecular diagnosis consistent with HLH: Presence of known HLH-related pathogenic gene mutations (e.g., pathological mutations in *PRF1, UNC13D, STX11, STXBP2, Rab27a, LYST, SH2D1A, BIRC4, ITK, AP3β1, MAGT1, CD27*, etc.). (2) Fulfillment of 5 or more of the following 8 criteria: (1) Fever: Temperature >38.5°C, lasting >7 days; (2) Splenomegaly; (3) Cytopenia (affecting two or three peripheral blood cell lineages): Hemoglobin <90 g/L (<100 g/L in infants <4 weeks), Platelets <100×10^9^/L, Neutrophils <1.0×10^9^/L, not attributable to reduced bone marrow hematopoiesis; (4) Hypertriglyceridemia and/or hypofibrinogenemia: Triglycerides >3 mmol/L or >3 standard deviations above age-specific norms, Fibrinogen <1.5 g/L or <3 standard deviations below age-specific norms; (5) Evidence of hemophagocytosis in bone marrow, spleen, liver, or lymph nodes; (6) Reduced or absent NK cell activity; (7) Elevated serum ferritin: Ferritin ≥500 μg/L; (8) Elevated soluble IL-2 receptor (sCD25) (12). It should be noted that the HLH-2004 diagnostic criteria, originally designed for pediatric populations, have demonstrated methodological constraints in adult clinical applications requiring further validation.

The primary endpoint, 30-day all-cause mortality, was defined as death ≤30 days post-admission.

### Statistical analysis

Baseline demographic data were summarized by presenting counts and percentages for categorical variables and reporting means (standard deviation) for normally distributed continuous variables or medians [minimum, maximum] for non-normally distributed variables. Analysis of variance (ANOVA) was used for normally distributed data, Wilcoxon rank-sum test for non-normally distributed data, and Chi-square test or Fisher’s exact test for categorical variables to compare baseline characteristics between groups (A: Infection-related, B: Malignancy-related, C: Autoimmune-related, D: Other). A *p*-value < 0.05 was considered statistically significant.

Trend line graphs were plotted, and temporal trends (case volume, diagnostics, treatments, 30-day mortality) were quantified using Sen’s slope estimator. Trend analysis was used to identify an appropriate time node for comparing patient data.

Independent risk factors were identified through Logistic regression, covariates with *p*<0.1 on univariate screening entered multivariable analysis. Variables with >10% missing data or high collinearity (variance inflation factor >5) were excluded. Model performance was evaluated using ROC-AUC analysis, with optimal cutoffs determined by the Youden index.

Kaplan-Meier curves compared 30-day survival distributions across treatment groups. Stratified log-rank tests assessed interactions between age and treatment modalities, underlying triggers and treatment modalities.

Resource utilization, namely length of hospital stay (LOS), was summarized as mean ± standard deviation.

### Software

Analyses used IBM SPSS version 23 and R software package version 4.3.

## Results

### Baseline characteristics

A total of 711 adult inpatients diagnosed with HLH were included in this study (2015–2023), comprising 424 males (59.6%) and 287 females (40.4%). The median age at diagnosis was 56 years (range: 18–88 years), with most patients (71.1%) falling between 43 and 78 years of age (**Supplementary Figure 1**). Diagnostic features aligned with the HLH-2004 criteria, as outlined in **Supplementary Table 1**.

At admission, hyperferritinemia (>500 µg/L) was observed in 91.4% of cases, with median ferritin concentration reaching 2596 µg/L (range: 16.9–18,450 µg/L). The predominant clinical symptom was fever (present in >90% of patients), followed by splenomegaly (40.9%) and lymphadenopathy (37.6%). EBV coinfection was detected in 43.3% of individuals, while infections (53.6%) emerged as the leading complication. Additional complications included coagulation disorders, multi-organ dysfunction, and hypoalbuminemia, with hepatic impairment (25.2%) being the most frequent organ-specific issue (**Table 1**).

**Table 1:** Baseline characteristics of HLH patients.

Characteristics	Total	I-HLH	M-HLH	A-HLH	Others	p.value
	N = 711	N = 221	N = 326	N = 78	N = 86	
Age,y	56.0 [18.0;88.0]	58.0 [18.0;87.0]	57.0 [18.0;88.0]	52.0 [22.0;83.0]	49.5 [18.0;82.0]	<0.001*
Sex						<0.001*
Male	424 (59.6%)	131 (59.3%)	227 (69.6%)	22 (28.2%)	44 (51.2%)	
Female	287 (40.4%)	90 (40.7%)	99 (30.4%)	56 (71.8%)	42 (48.8%)	
Mortality	241 (33.9%)	73 (33.0%)	128 (39.3%)	21 (26.9%)	19 (22.1%)	0.010*
Clinical features						
Tmax,°C	39.0 [36.0;43.0]	39.0 [36.0;42.0]	38.9 [36.0;43.0]	39.0 [36.0;42.0]	39.0 [36.2;42.0]	0.087
Hepatomegaly	63 (8.94%)	19 (8.68%)	36 (11.1%)	4 (5.13%)	4 (4.76%)	0.165
Splenomegaly	288 (40.9%)	76 (34.7%)	151 (46.6%)	26 (33.3%)	35 (41.7%)	0.021*
Lymphadenopathy	265 (37.6%)	59 (26.9%)	154 (47.5%)	27 (34.6%)	25 (29.8%)	<0.001*
Rash	96 (13.6%)	36 (16.4%)	37 (11.4%)	18 (23.1%)	5 (5.95%)	0.005*
Jaundice	45 (6.38%)	8 (3.65%)	24 (7.41%)	5 (6.41%)	8 (9.52%)	0.164
Edema	106 (15.1%)	26 (11.9%)	57 (17.6%)	11 (14.1%)	12 (14.5%)	0.328
Neurological	145 (20.6%)	54 (24.7%)	68 (21.0%)	10 (12.8%)	13 (15.5%)	0.091
Hemophagy	230 (32.6%)	86 (39.3%)	97 (29.9%)	20 (25.6%)	27 (32.1%)	0.066
Laboratory data						
CMV (+)	37 (5.45%)	16 (7.73%)	14 (4.44%)	6 (7.89%)	1 (1.23%)	0.083
EBV (+)	298 (43.2%)	133 (62.1%)	142 (44.7%)	20 (26.7%)	3 (3.61%)	<0.001*
HBV (+)	54 (8.91%)	12 (6.74%)	34 (11.5%)	3 (4.84%)	5 (7.14%)	0.173
WBC (10^9^/L)	3.69 [0.03;485]	3.88 [0.04;45.5]	3.30 [0.03;485]	4.85 [0.75;37.4]	3.98 [0.54;54.8]	<0.001*
LY (10^9^/L)	0.66 [0.00;41.1]	0.69 [0.02;6.01]	0.60 [0.01;41.1]	0.78 [0.00;3.09]	0.80 [0.10;6.17]	0.061
MO (10^9^/L)	0.29 [0.00;434]	0.26 [0.00;3.87]	0.28 [0.00;434]	0.38 [0.02;1.76]	0.36 [0.00;3.60]	0.102
ANC (10^9^/L)	2.34 [0.00;62.8]	2.52 [0.00;37.7]	2.07 [0.00;62.8]	3.54 [0.02;34.9]	2.47 [0.12;48.3]	<0.001*
LY (%)	18.9 [0.00;100]	18.2 [0.70;100]	19.8 [0.00;100]	13.5 [0.00;93.1]	18.9 [0.30;75.6]	0.027*
MO (%)	7.40 [0.00;89.6]	6.85 [0.00;48.0]	8.65 [0.00;89.6]	6.15 [0.50;30.9]	7.00 [0.00;48.3]	<0.001*
NE (%)	70.2 [0.00;98.5]	73.1 [0.00;96.6]	67.7 [0.00;97.7]	75.8 [2.00;98.5]	70.2 [0.01;95.9]	<0.001*
RBC (10^9^/L)	3.21 (0.84)	3.40 (0.86)	3.06 (0.80)	3.28 (0.79)	3.21 (0.89)	<0.001*
HGB (g/L)	92.0 [6.00;162]	97.5 [37.0;162]	88.0 [39.0;159]	90.0 [52.0;140]	90.5 [6.00;139]	<0.001*
PLT (10^9^/L)	59.5 [0.00;467]	60.0 [2.00;429]	48.0 [0.00;451]	103 [4.00;467]	70.5 [4.00;447]	<0.001*
PT (s)	13.4 [10.1;86.9]	13.5 [10.1;29.1]	13.6 [10.4;53.3]	13.0 [10.2;60.2]	13.2 [10.7;86.9]	0.096
INR	1.17 [0.88;7.63]	1.17 [0.88;2.52]	1.19 [0.92;5.16]	1.13 [0.89;5.23]	1.15 [0.93;7.63]	0.070
APTT (s)	34.2 [16.6;180]	35.0 [22.2;180]	34.8 [20.8;110]	30.5 [20.8;180]	32.2 [16.6;112]	<0.001*
FIB (g/L)	2.13 [0.21;10.2]	2.14 [0.23;10.2]	2.16 [0.29;9.48]	2.01 [0.21;8.03]	2.36 [0.61;9.20]	0.411
TT (s)	18.6 [13.9;120]	18.8 [14.3;120]	18.4 [13.9;120]	18.6 [14.8;54.6]	18.0 [14.7;37.5]	0.184
D-Dimer (mg/L)	3.05 [0.10;68.5]	3.24 [0.16;40.0]	2.86 [0.10;40.0]	4.13 [0.19;40.0]	2.43 [0.10;68.5]	0.068
ALT (U/L)	47.2 [2.00;3249]	58.5 [2.00;2572]	40.7 [2.90;3249]	46.4 [7.40;2894]	56.0 [8.20;452]	0.018*
AST (U/L)	64.8 [5.70;4688]	80.0 [6.90;4688]	59.3 [5.70;3568]	71.3 [10.2;2078]	52.3 [7.70;1679]	0.022*
ALP (U/L)	134 [19.0;1370]	110 [19.0;1370]	169 [19.8;1315]	114 [36.0;759]	101 [35.6;1002]	<0.001*
GGT (U/L)	85.9 [2.05;2174]	84.0 [2.05;2174]	91.8 [8.80;1410]	98.8 [7.90;1057]	71.7 [10.3;927]	0.160
LDH (U/L)	558 [85.0;10262]	530 [136,8558]	614 [85.0;10262]	598 [114,9425]	482 [103;6427]	0.422
CK (U/L)	29.0 [2.00;9610]	33.0 [2.00;9610]	27.0 [3.00;1778]	29.0 [4.00;1497]	24.4 [3.00;4984]	0.003*
HBDH (U/L)	348 [1.92;5505]	320 [1.92;3753]	388 [62.0;5505]	374 [24.6;2671]	335 [73.0;4865]	0.172
TB (µmol/L)	15.1 [0.21;541]	13.7 [4.10;452]	16.9 [0.21;443]	14.2 [4.50;541]	12.6 [4.66;388]	0.004*
DB (µmol/L)	7.30 [0.90;365]	6.10 [0.90;351]	8.65 [0.97;308]	6.75 [1.40;365]	5.70 [1.50;278]	0.004*
IB (µmol/L)	7.60 [0.22;177]	7.30 [1.80;120]	8.40 [0.22;138]	6.90 [2.80;177]	6.85 [1.71;154]	0.036*
TC (mmol/L)	3.15 [0.38;11.4]	3.05 [0.59;11.4]	3.06 [0.38;9.17]	3.63 [0.40;10.3]	3.37 [1.09;8.76]	0.003*
TG (mmol/L)	1.90 [0.35;14.9]	1.83 [0.42;14.9]	2.00 [0.41;10.5]	1.82 [0.35;11.8]	1.81 [0.67;9.26]	0.746
HDL-C (mmol/L)	0.59 [0.12;2.05]	0.62 [0.14;1.71]	0.51 [0.12;2.05]	0.73 [0.20;1.75]	0.70 [0.23;2.02]	<0.001*
LDL-C (mmol/L)	2.10 [0.52;6.82]	2.08 [0.52;6.82]	1.98 [0.64;6.73]	2.33 [1.04;5.78]	2.25 [0.61;6.16]	0.005*
LPa (mg/L)	68.0 [0.00;1146]	67.0 [0.00;1146]	65.0 [1.00;1119]	70.0 [3.00;953]	70.0 [2.00;961]	0.601
TP (g/L)	54.7 [25.6;130]	55.7 [25.6;76.4]	53.1 [34.4;78.4]	58.7 [40.3;81.6]	54.7 [38.8;130]	<0.001*
ALB (g/L)	29.1 (5.34)	29.3 (5.65)	28.8 (5.14)	29.3 (4.87)	29.6 (5.68)	0.460
A/G	1.20 [0.20;3.00]	1.10 [0.50;2.50]	1.20 [0.50;3.00]	0.95 [0.50;2.10]	1.20 [0.20;2.10]	<0.001*
GLU (mmol/L)	5.70 [1.40;26.3]	5.89 [1.40;23.1]	5.74 [2.29;20.5]	5.64 [2.43;26.3]	5.32 [2.48;16.2]	0.155
Urea (mmol/L)	5.76 [0.75;61.1]	5.78 [1.40;61.1]	6.26 [0.75;34.0]	5.48 [2.23;29.5]	4.88 [1.20;32.4]	0.001 *
Cr (µmol/L)	59.7 [21.7;562]	61.0 [22.1;562]	62.6 [21.7;554]	47.1 [25.1;403]	59.9 [33.7;363]	<0.001*
UA (µmol/L)	232 [4.23;1142]	223 [4.23;785]	249 [52.6;1121]	207 [30.4;650]	230 [28.0;1142]	0.012*
Ca (mmol/L)	2.01 [1.41;4.28]	2.00 [1.41;3.26]	2.01 [1.53;4.28]	2.06 [1.66;2.47]	2.01 [1.47;2.36]	0.149
Phos (mmol/L)	1.06 [0.25;2.68]	1.00 [0.25;2.08]	1.07 [0.27;2.26]	1.10 [0.36;1.89]	1.10 [0.28;2.68]	0.199
Mg (mmol/L)	0.86 [0.42;1.41]	0.88 [0.42;1.31]	0.84 [0.43;1.41]	0.84 [0.67;1.37]	0.85 [0.48;0.99]	0.012*
K (mmol/L)	3.73 [2.03;6.00]	3.70 [2.47;6.00]	3.80 [2.03;5.92]	3.65 [2.40;5.42]	3.74 [2.85;4.76]	0.083
Na (mmol/L)	136 [112;161]	136 [112;161]	136 [120;151]	138 [128;147]	137 [127;156]	0.003*
Cl (mmol/L)	102 [79.4;127]	101 [79.4;127]	102 [86.8;117]	102 [91.0;113]	103 [91.0;115]	0.149
RBP (mg/L)	20.3 [0.80;107]	21.5 [1.82;107]	18.7 [0.80;75.6]	19.9 [4.80;93.2]	21.7 [1.80;65.4]	0.403
ADA (U/L)	45.4 [1.50;575]	38.9 [5.20;575]	52.6 [1.50;437]	42.1 [10.0;226]	39.3 [3.60;329]	0.017 *
Ferritin (µg/L)	2596 [16.9;18450]	3210 [143;18450]	2569 [19.1;18450]	4354 [100;18450]	1410 [16.9;18450]	<0.001 *
IL-6 (pg/ml)	27.2 [0.02;11981]	36.2 [0.14;11981]	33.9 [0.04;8190]	14.7 [0.02;318]	13.2 [0.16;550]	0.069
CRP (mg/L)	43.8 [1.00;470]	51.3 [1.00;470]	42.6 [1.00;375]	46.0 [1.00;198]	47.2 [1.73;231]	0.820
sCD25 (ng/L)	18484 [0.00;280228]	16856 [0.00;147845]	25816 [787;280228]	13384 [645;80470]	18522 [2465;77401]	0.002 *
PCT (ng/ml)	0.35 [0.00;100]	0.32 [0.00;100]	0.38 [0.01;68.4]	0.23 [0.01;31.3]	0.37 [0.01;10.2]	0.266
NK cell (%)	5.90 [0.10;87.4]	7.40 [0.10;87.4]	6.30 [0.10;79.3]	3.60 [0.30;22.3]	5.30 [0.36;75.1]	<0.001*
Comorbidity						
EBV infection	308 (43.3%)	139 (62.9%)	146 (44.8%)	20 (25.6%)	3 (3.49%)	<0.001 *
Malignancy	353 (49.6%)	17 (7.69%)	324 (99.4%)	3 (3.85%)	9 (10.5%)	<0.001*
Autoimmune disease	103 (14.5%)	10 (4.52%)	12 (3.68%)	77 (98.7%)	4 (4.65%)	<0.001*
Lung disease	99 (13.9%)	47 (21.3%)	29 (8.90%)	16 (20.5%)	7 (8.14%)	<0.001*
Digestive disease	27 (3.80%)	9 (4.07%)	10 (3.07%)	5 (6.41%)	3 (3.49%)	0.528
Gallbladder disease	46 (6.47%)	14 (6.33%)	16 (4.91%)	8 (10.3%)	8 (9.30%)	0.230
Hepatopathy	78 (11.0%)	27 (12.2%)	33 (10.1%)	10 (12.8%)	8 (9.30%)	0.775
Kidney disease	38 (5.34%)	14 (6.33%)	15 (4.60%)	4 (5.13%)	5 (5.81%)	0.809
Hypothyroidism	24 (3.38%)	9 (4.07%)	10 (3.07%)	4 (5.13%)	1 (1.16%)	0.480
Angiopathy	27 (3.80%)	7 (3.17%)	13 (3.99%)	5 (6.41%)	2 (2.33%)	0.554
Postoperation	61 (8.58%)	15 (6.79%)	23 (7.06%)	11 (14.1%)	12 (14.0%)	0.045 *
Arrhythmia	45 (6.33%)	13 (5.88%)	20 (6.13%)	11 (14.1%)	1 (1.16%)	0.010 *
Heart failure	28 (3.94%)	11 (4.98%)	11 (3.37%)	3 (3.85%)	3 (3.49%)	0.808
Coronary heart disease	16 (2.25%)	5 (2.26%)	10 (3.07%)	0 (0.00%)	1 (1.16%)	0.479
Hypertension	114 (16.0%)	36 (16.3%)	56 (17.2%)	9 (11.5%)	13 (15.1%)	0.670
Cerebral infarction	31 (4.36%)	11 (4.98%)	13 (3.99%)	6 (7.69%)	1 (1.16%)	0.209
Diabetes	89 (12.5%)	31 (14.0%)	38 (11.7%)	8 (10.3%)	12 (14.0%)	0.751
Complication						
Lung infection	210 (29.5%)	61 (27.6%)	109 (33.4%)	23 (29.5%)	17 (19.8%)	0.081
Other infection	171 (24.1%)	41 (18.6%)	88 (27.0%)	22 (28.2%)	20 (23.3%)	0.113
Bleeding	47 (6.61%)	12 (5.43%)	26 (7.98%)	5 (6.41%)	4 (4.65%)	0.568
Abnormal coagulation	86 (12.1%)	24 (10.9%)	43 (13.2%)	8 (10.3%)	11 (12.8%)	0.808
Hypohepatia	179 (25.2%)	54 (24.4%)	79 (24.2%)	20 (25.6%)	26 (30.2%)	0.707
Renal insufficiency	43 (6.05%)	9 (4.07%)	27 (8.28%)	3 (3.85%)	4 (4.65%)	0.188
Cardiac damage	46 (6.47%)	17 (7.69%)	21 (6.44%)	4 (5.13%)	4 (4.65%)	0.741
Respiratory failure	65 (9.14%)	22 (9.95%)	31 (9.51%)	7 (8.97%)	5 (5.81%)	0.711
Shock	34 (4.78%)	14 (6.33%)	14 (4.29%)	5 (6.41%)	1 (1.16%)	0.201
MODS	25 (3.52%)	10 (4.52%)	11 (3.37%)	1 (1.28%)	3 (3.49%)	0.675
DIC	20 (2.82%)	3 (1.36%)	14 (4.29%)	2 (2.56%)	1 (1.16%)	0.188
Myelosuppression	82 (11.5%)	12 (5.43%)	62 (19.0%)	2 (2.56%)	6 (6.98%)	<0.001 *
Dropsy of serous cavity	70 (9.85%)	24 (10.9%)	31 (9.51%)	5 (6.41%)	10 (11.6%)	0.650
Hypoproteinemia	125 (17.6%)	40 (18.1%)	55 (16.9%)	15 (19.2%)	15 (17.4%)	0.960
Electrolyte disturbance	113 (15.9%)	35 (15.8%)	49 (15.0%)	12 (15.4%)	17 (19.8%)	0.762

I-HLH, Infection-related HLH; M-HLH, Malignancy-related HLH; A-HLH, Autoimmune-related HLH; EBV, Epstein Barr virus; CMV, Cytomegalo virus; HBV, Hepatitis B virus; WBC, White blood cell; LY, Lymphocyte count; MO, monocyte count; ANC, Neutrophil count; NE, Neutrophil; RBC, Red blood cell count; HGB, Hemoglobin; PLT, Platelet count; PT, Prothrombin time; INR, International normalized ratio; APTT, Activated partial thromboplastin time; TT, Thrombin time; FIB, Fibrinogen; ALT, Alanine aminotransferase; AST, Aspartate aminotransferase; ALP, Alkaline phosphatase; GGT, L-γ-glutamyl transpeptidase; LDH, Lactate dehydrogenase; CK, Creatine kinase; HBDH, α-hydroxybutyrate dehydrogenase; TB, Total bilirubin; DB, Direct bilirubin; IB, Indirect bilirubin; TC, Total cholesterol; TG, Triglycerides; HDL-C, High-density lipoprotein cholesterol; LDL-C, Low-density lipoprotein cholesterol; LPa, Lipoprotein a; TP, Total protein; ALB, Albumin; A/G, Albumin/Globulin ratio; Urea, Blood urea nitrogen; Cr, Creatinine; UA, Uric acid; Glu, Glucose; Ca, Calcium; Phos, Phosphorus; Mg, Magnesium; Cl, Chloride; Na, Sodium; RBP, Retinol-binding protein; ADA, Adenosine deaminase; IL-6, Interleukin-6; CRP, C-reactive protein; sCD25, Soluble interleukin-2 receptor α subunit; PCT, Procalcitonin; NK cell, Proportion of NK cells; DIC, Disseminated Intravascular Coagulation; MODS, Multiple organ dysfunction syndrome. *There is a large deficiency of IL-6, CRP, sCD25, PCT and NK cell.

### Trigger factors

Malignancy emerged as the predominant trigger (45.9%, n=326) in our cohort, followed by infectious causes (31.1%, n=221), autoimmune disorders (11.0%, n=78), and unidentified/other etiologies (12.1%, n=86). Among malignant triggers, non-Hodgkin lymphoma (NHL) was most prevalent, with T-cell lymphomas outnumbering B-cell subtypes (103 vs. 84; *p*< 0.05). No definitive trigger was detected in 9.0% (n=64) of cases.

Viral infections accounted for 25.3% of infectious triggers, with EBV being the leading viral pathogen (19.4%). Detailed frequencies of all triggers are presented in **Table 2**. Notable sex-based differences were observed: males exhibited higher malignancy rates (53.5%) than females (34.5%), while autoimmune diseases were more common in females (19.5% vs 5.2%). Age-stratified analysis revealed that patients under 56 years had higher rates of autoimmune conditions (13.3% vs 8.7%), more frequent unknown/other causes (15.8% vs 8.0%) and lower infection rates (27.4% vs 34.7%) compared to older patients (≥56 years). Complete subgroup analyses are available in **Supplementary Table 2**.

**Table 2:** List of triggers.

List of triggers
Malignancy: 326 (45.9%)
Hematologic: 319 (44.9%)
Non-Hodgkin Lymphomas (NHL): 270 (38.0%)
B-cell NHL: 84 (11.8%)
T-cell NHL: 103 (14.5%)
Unspecified NHL: 78 (11.0%)
Hodgkin Lymphomas: 7 (1.0%)
Leukemia: 40 (5.6%)
Lymphoid leukemias: 18 (2.5%)
Myeloid leukemias: 8 (1. 1%)
Myeloproliferative diseases: 14 (2.0%)
Plasma cell dyscrasias: 1 (0 1%)
Solid: 14 (2.0%)
Infections: 221 (31. 1%)
Viruses: 180 (25.3%)
EBV: 138 (19.4%)
CMV: 14 (2.0%)
Respiratory virus: 5 (0.7%)
Bunyaviridae: 16 (2.3%)
Viral hepatitis: 4 (0.6%)
HIV: 3 (0.4%)
Others: 4 (0.6%)
Bacteria: 60 (8.4%)
Gram-positive bacteria: 13 (1.8%)
Gram-negative bacteria: 7 (1.0%)
Mycobacteriun tuberculosis: 5 (0.7%)
Other or unspecified bacteria: 36 (5. 1%)
Fungus: 8 (1. 1%)
Others: 11 (1.5%)
Atypical pathogen: 3 (0.4%)
Schistosome: 3 (0.4%)
Unspecified: 5 (0.7%)
Autoimmune diseases: 78 (11.0%)
Connective tissue diseases (CTD): 67 (9.4%)
SLE: 22 (3. 1%)
Rheumatoid arthritis: 3 (0.4%)
Vasculitis: 4 (0.6%)
Siccasydrome:7 (1.0%)
Myositis: 10 (1.4%)
AOSD: 7(1.0%)
Others: 10 (1.4%)
Allergic inflammation: 11 (1.5%)
Other conditions: 86 (12. 1%)
Transplant: 7 (1.0%)
Gestation: 4 (0.6%)
No or unknown trigger: 64 (9.0%)
Others: 5 (0.7%)

NHL, Non-Hodgkin Lymphomas; EBV, Epstein Barr virus; CMV, Cytomegalo virus; HIV, Human immunodeficiency virus; SLE, Systemic lupus erythematosus; AOSD, Adult-Onset Still’s Disease; CML, Chronic myeloid leukemia. is counted in myeloid leukemias. In the Infection-related group, some cases had multiple pathogens overlapping.

### Trend analysis

**Figure 1:**
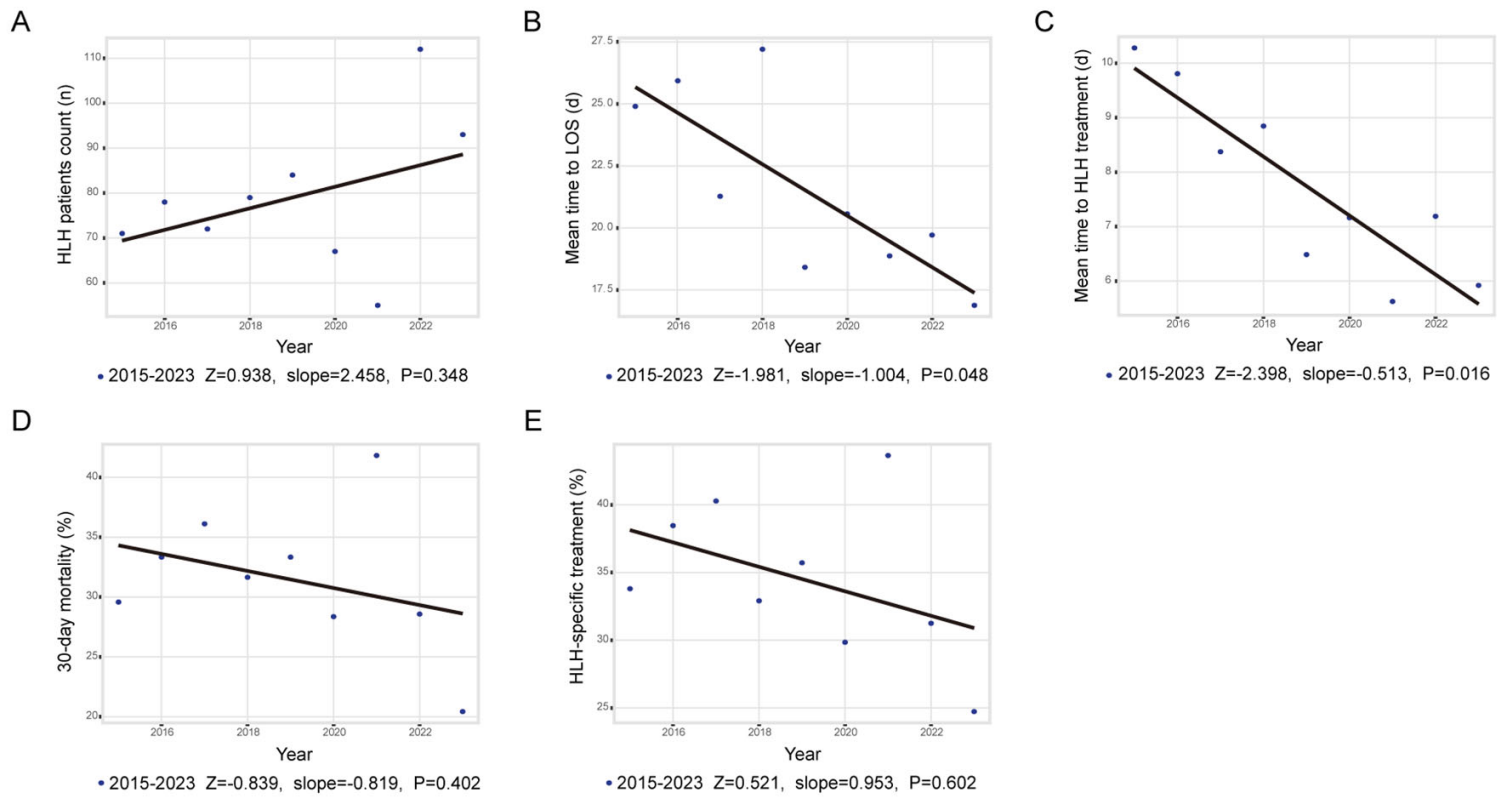
Trends and annual percentage changes. **(A)** Frequency of adult HLH cases; **(B)** Mean time to LOS; **(C)** Mean time to in-hospital treatment; **(D)** Rate of 30-day mortality; **(E)** Rate of in hospital HLH treatment.

**Figure 1a** demonstrates the annual fluctuations in patient admissions. While an upward trend in HLH incidence was noted during the study period (peak increase: 103.6%; Z = 0.938; slope = 2.458; *p* = 0.348), this trend did not achieve statistical significance. Admission rates remained stable from 2015 to 2019 (ADF test: *p*<0.05), decreased during 2020–2021, and rose markedly in 2022–2023.

As shown in **Figures 1b, c**, both length of stay (LOS; Z = -1.981, slope = -1.004, *p* = 0.048) and the average time from admission to initiation of HLH-specific therapy (Z = -2.398, slope = -0.513, *p* = 0.016) decreased significantly. **Figures 1d, e** depict annual changes in 30-day mortality (mean mortality: 31.5%; Z = -0.839, slope = -0.819, *p* = 0.402) and administration rate of HLH-specific therapy (Z = 0.521, slope = 0.953, *p* = 0.602). Neither indicator exhibited significant changes between 2015 and 2023. Subgroup analysis stratified by trigger factors revealed divergent mortality trends; however, none reached statistical significance (**Supplementary Figure 2**).

No significant differences were observed in trigger distributions (*p* = 0.790) when comparing periods before and after January 1, 2020 (designated as the pandemic onset reference), however, post-pandemic elevations occurred in inflammatory markers (white blood cell count (WBC), neutrophils, CRP, IL-6, PCT), comorbidities (pulmonary disease (18.3% vs. 10.2%; *p* = 0.002), arrhythmia (8.87% vs. 4.17%; *p* = 0.016)) and overall complications, particularly concurrent infections (31.7% vs. 17.2%; *p*< 0.001), though pulmonary infections remained unchanged (**Supplementary Table 3**).

### Subgroup analysis

**Figure 2:**
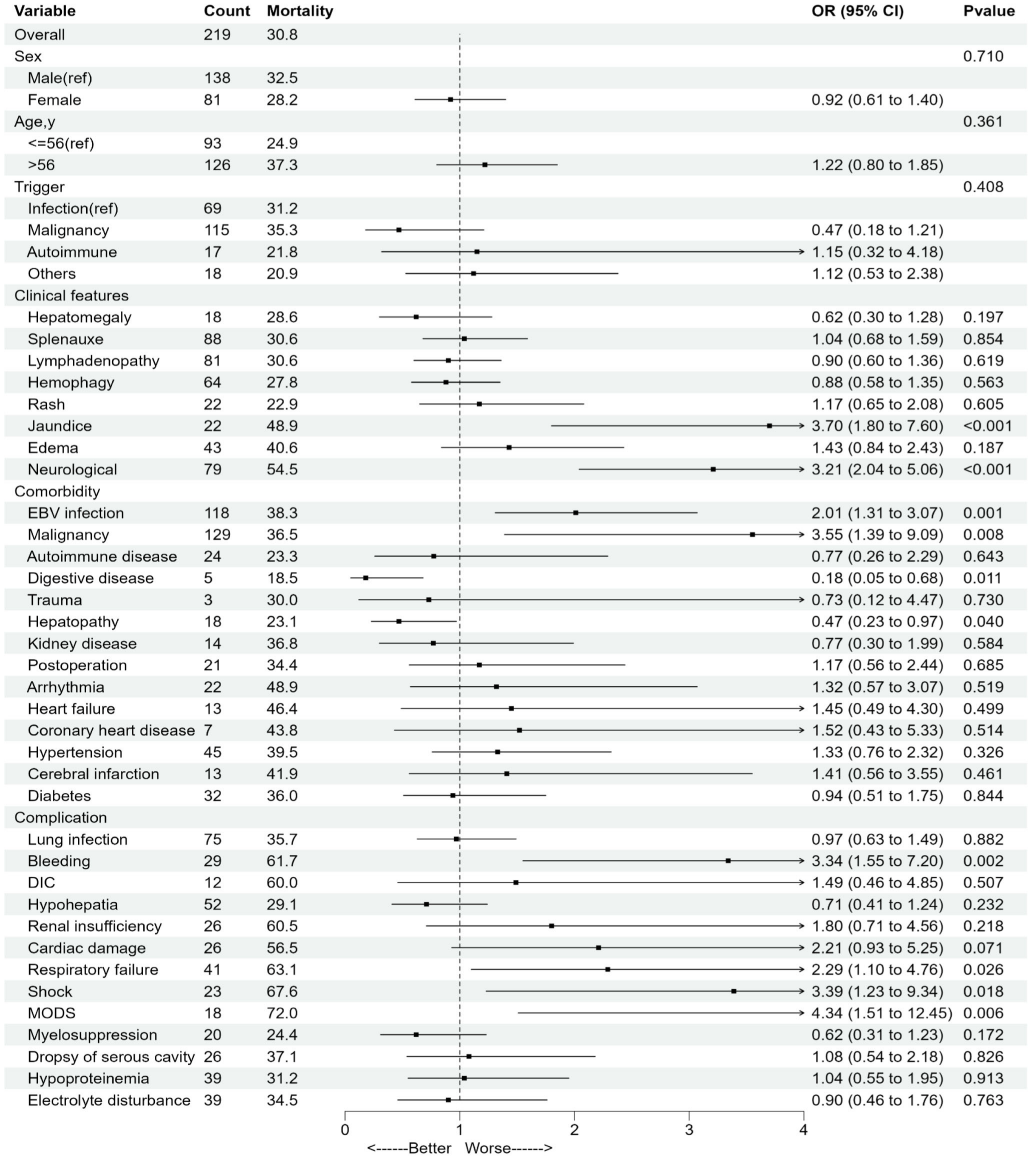
30-day mortality rates and adjusted odds for different associated conditions. Digestive disease includes Gastrointestinal polyps, Gastroenteritis, Esophagitis and Peptic ulcer. Hepatopathy includes Viral hepatitis, Steatohepatitis, Alcoholic hepatitis and Hepatic cyst. OR, odds ratio; CI, confidence interval.

**Figure 2** presents 30-day mortality rates stratified by demographic and related condition subgroups. The leading causes of death were progression of hematologic malignancies (37.44%), infectious complications (28.31%), organ failure (12.79%) (**Supplementary Figure 3**).

### Prediction of 30-day prognosis

Univariate analysis indicated significantly elevated concentration of LY, MO, RBC, HGB, PLT, FIB, HDL-C, LPa, TP, ALB, Ca, Phos, Na and RBP in survivors versus non-survivors (all *p*<0.05). Conversely, survivors exhibited significantly lower values for age, PT, INR, APTT, TT, D-Dimer, ALT, AST, ALP, GGT, LDH, HBDH, TB, DB, IB, TG, Urea, Cr, Glu, ADA, sCD25, PCT and Ferritin (all *p*<0.05). Multivariate logistic regression identified seven independent predictors: age (HR, 1.028; 95% CI, 1.013–1.043; *p*<0.001), ferritin (HR, 1.000; 95% CI, 1.000–1.000; *p*<0.001), APTT (HR, 1.024; 95% CI, 1.009–1.039; *p* = 0.001), ALT (HR, 1.001; 95% CI, 1.000–1.003; *p* = 0.009), BUN (HR, 1.105; 95% CI, 1.063–1.148; *p*<0.001), phosphorus (HR, 0.435; 95% CI, 0.226–0.822; *p* = 0.011), and chloride (HR, 0.959; 95% CI, 0.926–0.994; *p* = 0.021) (**Table 3**). A prognostic model for 30-day mortality was derived:

**Table 3:** Logistic regression analysis of independent factors and 30-day mortality.

Variables	Univariate analysis			Multivariate analysis			
	HR	95% CI	P.value	β	HR	95% CI	P.value
Age,y	1.023	1.012-1.034	<0.001	0.0273	1.028	1.013-1.043	<0.001
PLT (109/L)	0.993	0.990-0.995	<0.001				
APTT (s)	1.041	1.021-1.055	<0.001	0.0239	1.024	1.009-1.039	0.001
FIB (g/L)	0.769	0.683-0.866	<0.001				
TT (s)	1.050	1.029-1.072	<0.001				
D-Dimer (mg/L)	1.033	1.016-1.051	<0.001				
ALT (U/L)	1.001	1.000-1.002	0.004	0.0014	1.001	1.000-1.003	0.009
AST (U/L)	1.002	1.001-1.002	<0.001				
ALP (U/L)	1.002	1.001-1.002	<0.001				
CK (U/L)	1.000	1.000-1.000	0.957				
GGT (U/L)	1.002	1.001-1.003	<0.001				
TG (mmol/L)	1.332	1.197-1.481	<0.001				
HDL-C (mmol/L)	0.289	0.162-0.515	<0.001				
LPa (mg/L)	0.998	0.997-0.999	0.005				
UA (mmol/L)	1.002	1.001-1.003	0.001				
GLU (mmol/L)	1.111	1.050-1.175	<0.001				
Urea (mmol/L)	1.113	1.079-1.047	<0.001	0.0995	1.105	1.063-1.148	<0.001
Cr (µmol/L)	1.008	1.005-1.011	<0.001				
Ca (mmol/L)	0.178	0.068-0.470	<0.001				
Phos (mmol/L)	0.591	0.353-0.990	0.046	-0.8411	0.431	0.226-0.822	0.011
K (mmol/L)	1.420	1.064-1.896	0.017				
Na (mmol/L)	0.969	0.940-0.999	0.043				
Cl (mmol/L)	0.973	0.945-1.002	0.072	-0.0417	0.959	0.926-0.994	0.021
Ferritin (µg/L)	1.000	1.000-1.000	<0.001	0.0001	1.000	1.000-1.000	<0.001

PLT, Platelet count; APTT, Activated partial thromboplastin time; TT, Thrombin time; FIB, Fibrinogen; ALT, Alanine aminotransferase; AST, Aspartate aminotransferase; ALP, Alkaline phosphatase; GGT, L-γ-glutamyl transpeptidase; CK, Creatine kinase; TG, Triglycerides; HDL-C, High-density lipoprotein cholesterol; LPa, Lipoprotein a; Urea, Blood urea nitrogen; Cr, Creatinine; UA, Uric acid; Glu, Glucose; Ca, Calcium; Phos, Phosphorus; Mg, Magnesium; Cl, Chloride; Na, Sodium.

Model:

Logit P = 0.0273 × age + 0.0239 × APTT + 0.0014 × ALT + 0.0995 × Urea - 0.8411 × phosphorus - 0.0417 × chloride + 0.0001 × Ferrin + 0.2360.

The ROC curve showed discriminatory power with an AUC of 0.781 (95% CI, 0.741–0.821; *p*<0.001). Using the Youden index, the optimal cutoff value was 0.298, yielding a sensitivity of 66.1% and specificity of 77.2% (**Supplementary Figure 4**).

### Treatment

**Figure 3:**
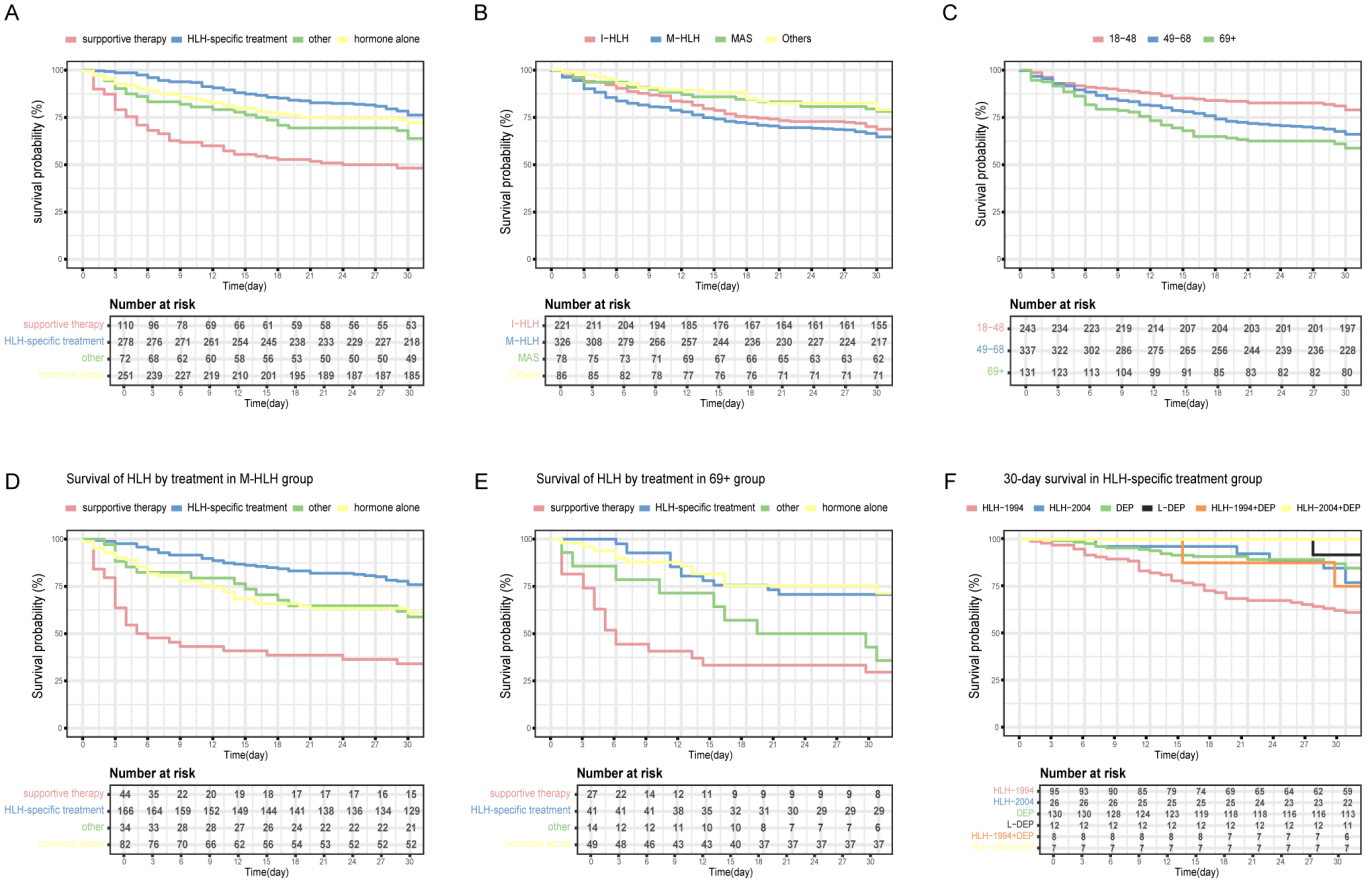
30-day survival estimates by treatment. **(A)** 30-day survival estimates by treatment; **(B)** 30-day survival estimates by triggers; **(C)** 30-day survival estimates by age; **(D)** 30-day survival of HLH by treatment in M-HLH group; **(E)** 30-day survival of HLH by treatment in 69+ age group; **(F)** 30-day survival in HLH-specific treatment group.

Among 711 patients with HLH, 39.1% (n=278) were treated with HLH-specific therapies (e.g., HLH-1994, DEP. all containing corticosteroids), whereas the majority (60.9%, n=433) received non-specific management, including corticosteroids, management of the underlying condition, or supportive care. The median survival for those who died within 30 days was 10 days. The overall 30-day survival rate was 69.2%, with significant variation by: treatment modality (supportive care, 48.2%; HLH-specific therapy, 78.4%; corticosteroid monotherapy, 73.7%; other, 68.1%, *p*< 0.001), age (18–48 years, 81.1%; 49–68 years, 67.7%; ≥69 years, 61.1%, *p*< 0.001), and trigger (infection, 70.1%; malignancy, 66.6%; autoimmune disease, 79.5%; other, 82.6%, *p* = 0.015) (**Figures 3a-c**). For malignancy-associated HLH, patients receiving HLH-specific therapy had markedly higher survival (77.7%) compared to alternative modalities (34.1–63.4%; *p*<0.001) (**Figure 3d**). In non-malignancy subgroups, for infectious, MAS, or other triggers, as well as in younger patients (18–68 years), treatment type (excluding supportive care) showed no significant survival difference (all *p*>0.05; **Supplementary Figure 5**). However, patients aged ≥69 years had worse outcomes if corticosteroids were omitted from their regimen (70.7% -75.5% vs. 29.6%–42.9%; *p*<0.001) (**Figure 3e**). Isolated supportive care consistently correlated with poor survival across all subgroups. Among HLH-specific protocols (HLH-2004, DEP, L-DEP, HLH-1994+DEP, HLH-2004+DEP), 30-day survival rates were comparable (*p* = 0.550) (**Figure 3f**).

### Resource utilization

The average hospital stay across all HLH cases was 21.4 days. When analyzed by etiology, autoimmune-associated HLH had the prolonged hospitalization duration (mean 26.6 days). In contrast, HLH linked to malignancies demonstrated the briefest mean LOS (20.0 days). Detailed LOS comparisons are presented in **Table 4**.

**Table 4:** Resource utilization.

Trigger	LOS, days
I-HLH (N = 221)	20.5(17.8)
M-HLH (N = 326)	20.0(17.6)
A-HLH (N = 76)	26.6(24.8)
Others (N = 86)	24.4(22.0)
Total (N = 711)	21.4(19.2)

I-HLH, Infection-related HLH; M-HLH, Malignancy-related HLH; A-HLH, Autoimmune-related HLH; LOS, Length of hospital stay.

## Discussion

HLH requires urgent intervention. In 711 Chinese adults (59.6% male; 71.1% aged 43–78), EBV triggered 19% of cases, while comorbidities occurred in 43%—higher than literature reports due to universal EBV screening (2, 10). Malignancy (46%), infection (31%), and autoimmunity (11%) were predominant triggers, aligning with US data (2) but contrasting with infection-dominant Chinese series (13), highlighting regional heterogeneity.

Incidence gradually increased until plateauing during 2020–2021 COVID-19 restrictions (14–16), rebounding post-2022. Earlier HLH therapy initiation (sometimes pre-diagnosis) and shorter LOS reflected improved awareness (17). Post-pandemic cases exhibited elevated inflammatory markers, possibly indicating SARS-CoV-2–associated hyperinflammation (18, 19).

Thirty-day mortality was highest in malignancy-associated HLH (36.5%), followed by infection (29.9%) and autoimmunity (20.5%) (20, 21), diverging from prior mixed-endpoint studies (22). Deaths primarily resulted from hematologic malignancy progression, refractory sepsis, or multi-organ failure. Age, ferritin (22–24), ALT, prolonged APTT (24, 25), BUN, hypochloremia (24, 26, 27), and novelly identified hypophosphatemia independently predicted 30-day mortality. As a conserved acute phase reactant, ferritin amplifies inflammation by stimulating key mediators (23, 28), with concentration >50,000 µg/L strongly predicting mortality (22). Although excluded from standard HLH criteria, elevated ALT (occurring in 83.6% of patients (11, 22)) independently predicts short-term outcomes. Prolonged APTT independently indicates 30-day mortality in adults, extending pediatric observations (25). Hypochloremia has been recognized as a marker of fluid imbalance associated with disease progression (26, 27). In addition, phosphorus dysregulation emerged as the strongest prognostic factor in our study, a finding not widely reported potentially linked to sepsis-related mechanisms (29).

Current first-line therapy mirrors pediatric HLH-1994/2004 (etoposide, steroids, calcineurin inhibitors) (12, 30, 31); Chinese guidelines also incorporate DEP (liposomal doxorubicin, etoposide, and methylprednisolone)/L-DEP (DEP plus pegaspargase) salvage (32, 33). Supportive care alone uniformly failed, likely due to advanced disease, prior treatment failure, or patient concerns regarding therapy costs/risks. The combination therapy cohort was primarily composed of refractory cases, likely explains why HLH-1994+DEP showed no advantage over monotherapy. Meanwhile, all HLH-2004+DEP patients survived likely due to small cohort. HLH-specific protocols showed no significant differences in early survival advantage except HLH-1994, indicating that complication management may outweighs regimen selection for early outcomes. Elderly (>69 y) showed a significant association between the lack of systemic corticosteroid and increased mortality, likely due to reduced cytokine storm tolerance. No mortality improvement over decades underscores the need for novel agents (anakinra, ruxolitinib, alemtuzumab, emapalumab), though large RCTs are lacking (34, 35).

Resource utilization analysis revealed the substantial healthcare burden of HLH management, with an average hospital stay of 21.4 days - 2.43 times longer than typical medical admissions in China.

This study’s key strengths include its large patient cohort and comprehensive analysis of comorbid conditions. To our knowledge, it is the first to assess the indirect impact of the COVID-19 pandemic on HLH epidemiology and hospitalization trends, rather than focusing solely on SARS-CoV-2’s direct effects. Additionally, we identified serum phosphorus as a novel independent prognostic factor in adult HLH and provided new insights into treatment outcomes. Limitations include retrospective single-center design (risk of selection bias), heterogeneous ‘other’ category, potential prior treatment confounders, and lack of long term outcome and genetic/inflammatory biomarkers (e.g., sCD25, IL-6) for refined prognostication. Future studies incorporating these biomarkers could improve HLH risk stratification.

In conclusion, this study analyzes the experience of a single center with the adult HLH population, which is associated with extensive clinical and laboratory findings as well as underlying diseases. The incidence of HLH continues to rise. Unfortunately, physicians still have insufficient awareness regarding the treatment of HLH in adult patients. The COVID - 19 pandemic has an indirect impact on HLH patients, which should alert physicians to the possibility of a potentially uncontrolled inflammatory state. Moreover, the identification of hypophosphatemia as an independent prognostic factor warrants further research. Therapeutic regimens for adult HLH patients require refinement to enhance prognostic outcomes. For instance, malignancy-associated HLH cases may benefit from HLH-specific therapeutic protocols, while geriatric patients could receive systemic corticosteroid management strategies. Our results underscore the imperative for optimized, adult-specific HLH management strategies.

## Data Availability

The original contributions presented in the study are included in the article/**Supplementary Material**. Further inquiries can be directed to the corresponding authors.
