# Supplementary material for: Epidemiological, clinical characteristics and prognostic factors analysis of adult patients with hemophagocytic lymphohistiocytosis in a Chinese hospital
Source: Front Immunol. 2025 Dec 12;16:1684308. doi: 10.3389/fimmu.2025.1684308 (PMC12740881; doi:10.3389/fimmu.2025.1684308)
Supplement: Supplementary file 1 [file DataSheet1.doc]

**Table of contents**

[Table S1 Clinical and laboratory findings according to the HLH-2004 Criteria 2](#__RefHeading___Toc10393)

[Table S2 Trigger in different subgroups of age and sex. 3](#__RefHeading___Toc29221)

[Table S3 The characteristics of the HLH patients before-and-after COVID-19 Pandemic. 4](#__RefHeading___Toc25517)

[Figure S1: Population pyramid showing patient distribution across different age and sex groups 8](#__RefHeading___Toc4928)

[Figure S2 30-day mortality trend for different triggers associated HLH. 9](#__RefHeading___Toc27633)

[Figure S3 Causes of adult HLH 30-day death. 10](#__RefHeading___Toc4044)

[Figure S4 Performance of the models. (A) Characteristic feature of the curve for model; (B) Calibration curves for 30-day mortality, which are indicative of predictive accuracy. 11](#__RefHeading___Toc11745)

[Figure S5 30-day survival estimates by treatment. 12](#__RefHeading___Toc15458)

[Figure S6 The treatment protocol outlined in the Chinese guide 13](#__RefHeading___Toc1427)

**Table S1 Clinical and laboratory findings according to the HLH-2004 Criteria**

| **Characteristics** | **No. (%) of patients** |
| --- | --- |
| Fever (t≥38.5℃) | 438/711(61.6) |
| Splenomegaly | 288/711 (40.5) |
| Bicytopenia or pancytopenia (ANC<1×10^9^ /L,hemoglobin<90 g/L, platelet count<100×109 /L) | 311/710 (47.3) |
| Hypertriglyceridemia (> 3.0 mmol/L) | 164/667 (22.6) |
| Hypofibrinogenemia (<1.5 g/L) | 196/706 (27.6) |
| Hemophagocytosis | 230/705 (23.6) |
| Hyperferritinemia (> 500 μg/L) | 631/690 (91.4) |
| Elevated soluble IL-2 receptor ( ≥2400 U/mL or≥6400 ng/L) | 158/181 (87.3) |
| Absent or decreased NK cell function | 13/24 (54.2) |

| **Trigger** | **sex** | | **age** | |
| --- | --- | --- | --- | --- |
|  | **male, n(%)** | **female, n(%)** | **≥56, n(%)** | **<56, n(%)** |
| I-HLH | 131(30.9) | 90(31.4) | 124(34.7) | 97(27.4) |
| M-HLH | 227(53.5) | 99(34.5) | 174(48.7) | 152(42.9) |
| A-HLH | 22(5.2) | 56(19.5) | 31(8.7) | 47(13.3) |
| Others | 44(10.4) | 42(14.6) | 28(7.8) | 58(16.4) |

**Table S2 Trigger in different subgroups of age and sex.**

**Table S3 The characteristics of the HLH patients before-and-after COVID-19 Pandemic.**

| **Characteristics** | **Total** | **Pre-Pandemic** | **Post-Pandemic** | **p.value** |
| --- | --- | --- | --- | --- |
|  | ***N=711*** | ***N=384*** | ***N=327*** |  |
| Age,y | 56.0 [18.0;88.0] | 55.0 [18.0;88.0] | 56.0 [18.0;87.0] | 0.230 |
| Sex |  |  |  | 0.490 |
| Male | 424 (59.6%) | 234 (60.9%) | 190 (58.1%) |  |
| Female | 287 (40.4%) | 150 (39.1%) | 137 (41.9%) |  |
| Mortality | 241 (33.9%) | 139 (36.2%) | 102 (31.2%) | 0.185 |
| Trigger |  |  |  | 0.790 |
| I-HLH | 221 (31.1%) | 120 (31.2%) | 101 (30.9%) |  |
| M-HLH | 326 (45.9%) | 178 (46.4%) | 148 (45.3%) |  |
| A-HLH | 78 (11.0%) | 38 (9.90%) | 40 (12.2%) |  |
| Others | 86 (12.1%) | 48 (12.5%) | 38 (11.6%) |  |
| Clinical features |  |  |  |  |
| Tmax,℃ | 39.0 [36.0;43.0] | 39.1 [36.4;42.0] | 38.0 [36.0;43.0] | <0.001* |
| Hepatomegaly | 63 (8.94%) | 48 (12.5%) | 15 (4.67%) | <0.001* |
| Splenomegaly | 288 (40.9%) | 189 (49.2%) | 99 (30.8%) | <0.001* |
| Lymphadenopathy | 265 (37.6%) | 155 (40.4%) | 110 (34.3%) | 0.113 |
| Rash | 96 (13.6%) | 62 (16.1%) | 34 (10.6%) | 0.042* |
| Jaundice | 45 (6.38%) | 38 (9.90%) | 7 (2.18%) | <0.001* |
| Edema | 106 (15.1%) | 61 (15.9%) | 45 (14.1%) | 0.570 |
| Neurological | 145 (20.6%) | 97 (25.3%) | 48 (15.0%) | 0.001* |
| Hemophagy | 230 (32.6%) | 115 (29.9%) | 115 (35.8%) | 0.115 |
| Laboratory data |  |  |  |  |
| CMV (+) | 37 (5.45%) | 8 (2.08%) | 29 (9.83%) | <0.001* |
| EBV (+) | 298 (43.2%) | 163 (42.4%) | 135 (44.1%) | 0.717 |
| HBV (+) | 54 (8.91%) | 23 (5.99%) | 31 (14.0%) | 0.002* |
| WBC (10^9^/L) | 3.69 [0.03;485] | 3.24 [0.04;117] | 4.43 [0.03;485] | <0.001* |
| LY (10^9^/L) | 0.66 [0.00;41.1] | 0.63 [0.01;41.1] | 0.73 [0.00;28.6] | 0.531 |
| MO (10^9^/L) | 0.29 [0.00;434] | 0.23 [0.00;12.2] | 0.37 [0.00;434] | <0.001* |
| ANC (10^9^/L) | 2.34 [0.00;62.8] | 2.03 [0.00;62.8] | 3.04 [0.01;37.7] | <0.001* |
| LY (%) | 18.9 [0.00;100] | 21.0 [0.70;100] | 16.4 [0.00;94.7] | 0.001* |
| MO (%) | 7.40 [0.00;89.6] | 7.25 [0.00;48.3] | 7.60 [0.00;89.6] | 0.520 |
| NE (%) | 70.2 [0.00;98.5] | 68.5 [0.00;97.7] | 73.5 [0.01;98.5] | 0.030* |
| RBC (10^9^/L) | 3.21 (0.84) | 3.27 (0.88) | 3.13 (0.78) | 0.022* |
| HGB (g/L) | 92.0 [6.00;162] | 94.0 [6.00;162] | 89.0 [39.0;156] | 0.122 |
| PLT (10^9^/L) | 59.5 [0.00;467] | 56.0 [2.00;409] | 66.0 [0.00;467] | 0.032* |
| PT (s) | 13.4 [10.1;86.9] | 13.7 [10.1;86.9] | 13.2 [10.4;53.3] | <0.001* |
| INR | 1.17 [0.88;7.63] | 1.19 [0.88;7.63] | 1.15 [0.90;5.16] | 0.001* |
| APTT (s) | 34.2 [16.6;180] | 36.3 [20.8;180] | 32.2 [16.6;110] | <0.001* |
| FIB (g/L) | 2.13 [0.21;10.2] | 2.18 [0.21;8.03] | 2.12 [0.54;10.2] | 0.246 |
| TT (s) | 18.6 [13.9;120] | 18.9 [14.8;120] | 18.1 [13.9;120] | <0.001* |
| D-Dimer (mg/L) | 3.05 [0.10;68.5] | 3.16 [0.10;68.5] | 2.99 [0.11;40.0] | 0.394 |
| ALT (U/L) | 47.2 [2.00;3249] | 53.6 [3.50;2572] | 38.2 [2.00;3249] | 0.001* |
| AST (U/L) | 64.8 [5.70;4688] | 71.3 [7.60;4688] | 55.2 [5.70;1966] | 0.032* |
| ALP (U/L) | 134 [19.0;1370] | 135 [19.0;1370] | 132 [19.8;1249] | 0.748 |
| GGT (U/L) | 85.9 [2.05;2174] | 83.3 [7.90;2174] | 94.7 [2.05;1156] | 0.298 |
| LDH (U/L) | 558 [85.0;10262] | 592 [97.0;9425] | 538 [85.0;10262] | 0.452 |
| CK (U/L) | 29.0 [2.00;9610] | 31.0 [2.00;7067] | 27.0 [3.00;9610] | 0.084 |
| HBDH (U/L) | 348 [1.92;5505] | 354 [69.0;5505] | 343 [1.92;3857] | 0.368 |
| TB (µmol/L) | 15.1 [0.21;541] | 15.6 [4.10;541] | 14.8 [0.21;350] | 0.347 |
| DB (µmol/L) | 7.30 [0.90;365] | 7.50 [1.40;365] | 6.90 [0.90;238] | 0.238 |
| IB (µmol/L) | 7.60 [0.22;177] | 7.80 [1.80;177] | 7.47 [0.22;120] | 0.343 |
| TC (mmol/L) | 3.15 [0.38;11.4] | 2.99 [0.38;11.4] | 3.34 [0.60;11.1] | 0.005* |
| TG (mmol/L) | 1.90 [0.35;14.9] | 1.88 [0.35;14.9] | 1.92 [0.41;12.0] | 0.580 |
| HDL-C (mmol/L) | 0.59 [0.12;2.05] | 0.57 [0.14;2.05] | 0.62 [0.12;1.92] | 0.013* |
| LDL-C (mmol/L) | 2.10 [0.52;6.82] | 2.12 [0.52;6.82] | 2.09 [0.61;6.56] | 0.116 |
| LPa (mg/L) | 68.0 [0.00;1146] | 69.0 [1.00;961] | 62.0 [0.00;1146] | 0.263 |
| ALB (g/L) | 29.1 (5.34) | 28.5 (5.46) | 29.7 (5.14) | 0.004* |
| GLU (mmol/L) | 5.70 [1.40;26.3] | 5.93 [2.29;24.2] | 5.27 [1.40;26.3] | <0.001* |
| Urea (mmol/L) | 5.76 [0.75;61.1] | 5.50 [1.20;61.1] | 6.26 [0.75;40.0] | 0.008* |
| Cr (µmol/L) | 59.7 [21.7;562] | 60.0 [24.1;562] | 58.7 [21.7;554] | 0.978 |
| UA (µmol/L) | 232 [4.23;1142] | 234 [28.0;1142] | 231 [4.23;796] | 0.796 |
| Ca (mmol/L) | 2.01 [1.41;4.28] | 2.00 [1.41;2.48] | 2.03 [1.53;4.28] | <0.001* |
| Phos (mmol/L) | 1.06 [0.25;2.68] | 1.05 [0.26;2.68] | 1.08 [0.25;2.26] | 0.784 |
| Mg (mmol/L) | 0.86 [0.42;1.41] | 0.87 [0.43;1.41] | 0.83 [0.42;1.17] | <0.001* |
| K (mmol/L) | 3.73 [2.03;6.00] | 3.75 [2.35;6.00] | 3.70 [2.03;5.92] | 0.199 |
| Na (mmol/L) | 136 [112;161] | 136 [120;159] | 137 [112;161] | 0.010* |
| Cl (mmol/L) | 102 [79.4;127] | 102 [88.0;126] | 102 [79.4;127] | 0.881 |
| ADA (U/L) | 45.4 [1.50;575] | 49.8 [1.50;437] | 38.9 [3.60;575] | 0.001* |
| Ferritin (µg/L) | 2596 [16.9;18450] | 2260 [19.1;18450] | 3052 [16.9;18450] | 0.035* |
| IL-6 (pg/ml) | 27.2 [0.02;11981] | 16.8 [0.02;1134] | 29.1 [0.04;11981] | 0.016* |
| CRP (mg/L) | 43.8 [1.00;470] | 41.1 [1.00;341] | 53.7 [1.17;470] | 0.006* |
| sCD25 (ng/L) | 18484 [0.00;280228] | 24540 [1077;70550] | 15881 [0.00;280228] | 0.019* |
| PCT (ng/ml) | 0.35 [0.00;100] | 0.38 [0.01;100] | 0.30 [0.00;68.4] | 0.010* |
| NK cell (%) | 5.90 [0.10;87.4] | 6.60 [0.10;87.4] | 5.40 [0.30;75.1] | 0.341 |
| Comorbidity |  |  |  |  |
| EBV infection | 308 (43.3%) | 163 (42.4%) | 145 (44.3%) | 0.666 |
| Malignancy | 353 (49.6%) | 187 (48.7%) | 166 (50.8%) | 0.635 |
| Autoimmune/Rheumatologic | 103 (14.5%) | 48 (12.5%) | 55 (16.8%) | 0.127 |
| Lung disease | 99 (13.9%) | 39 (10.2%) | 60 (18.3%) | 0.002* |
| Angiopathy | 27 (3.80%) | 7 (1.82%) | 20 (6.12%) | 0.005* |
| Postoperation | 61 (8.58%) | 31 (8.07%) | 30 (9.17%) | 0.698 |
| Arrhythmia | 45 (6.33%) | 16 (4.17%) | 29 (8.87%) | 0.016* |
| Complication |  |  |  |  |
| Lung infection | 210 (29.5%) | 116 (30.2%) | 94 (28.7%) | 0.731 |
| Other infection | 171 (24.1%) | 66 (17.2%) | 105 (32.1%) | <0.001* |
| Bleeding | 47 (6.61%) | 16 (4.17%) | 31 (9.48%) | 0.007* |
| Abnormal coagulation | 86 (12.1%) | 9 (2.34%) | 77 (23.5%) | <0.001* |
| Hypohepatia | 179 (25.2%) | 25 (6.51%) | 154 (47.1%) | <0.001* |
| Renal insufficiency | 43 (6.05%) | 8 (2.08%) | 35 (10.7%) | <0.001* |
| Cardiac damage | 46 (6.47%) | 14 (3.65%) | 32 (9.79%) | 0.002* |
| Respiratory failure | 65 (9.14%) | 24 (6.25%) | 41 (12.5%) | 0.006* |
| Shock | 34 (4.78%) | 12 (3.12%) | 22 (6.73%) | 0.039* |
| MODS | 25 (3.52%) | 11 (2.86%) | 14 (4.28%) | 0.413 |
| DIC | 20 (2.82%) | 7 (1.82%) | 13 (3.99%) | 0.131 |
| Myelosuppression | 82 (11.5%) | 11 (2.86%) | 71 (21.7%) | <0.001* |
| Dropsy of serous cavity | 70 (9.85%) | 15 (3.91%) | 55 (16.8%) | <0.001* |
| Hypoproteinemia | 125 (17.6%) | 18 (4.69%) | 107 (32.7%) | <0.001* |
| Electrolyte disturbance | 113 (15.9%) | 19 (4.95%) | 94 (28.7%) | <0.001* |


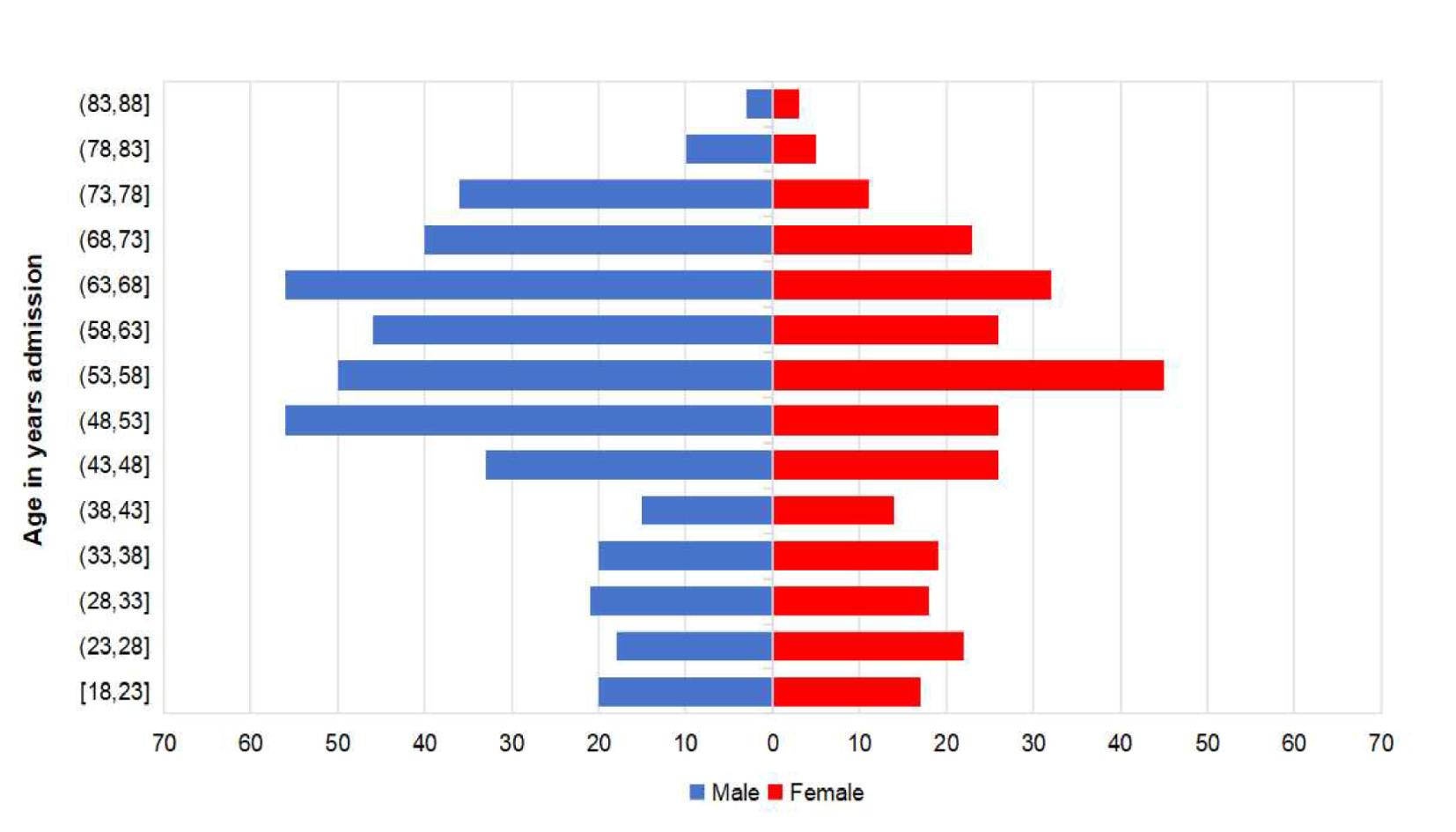


**Figure S1: Population pyramid showing patient distribution across different age and sex groups**


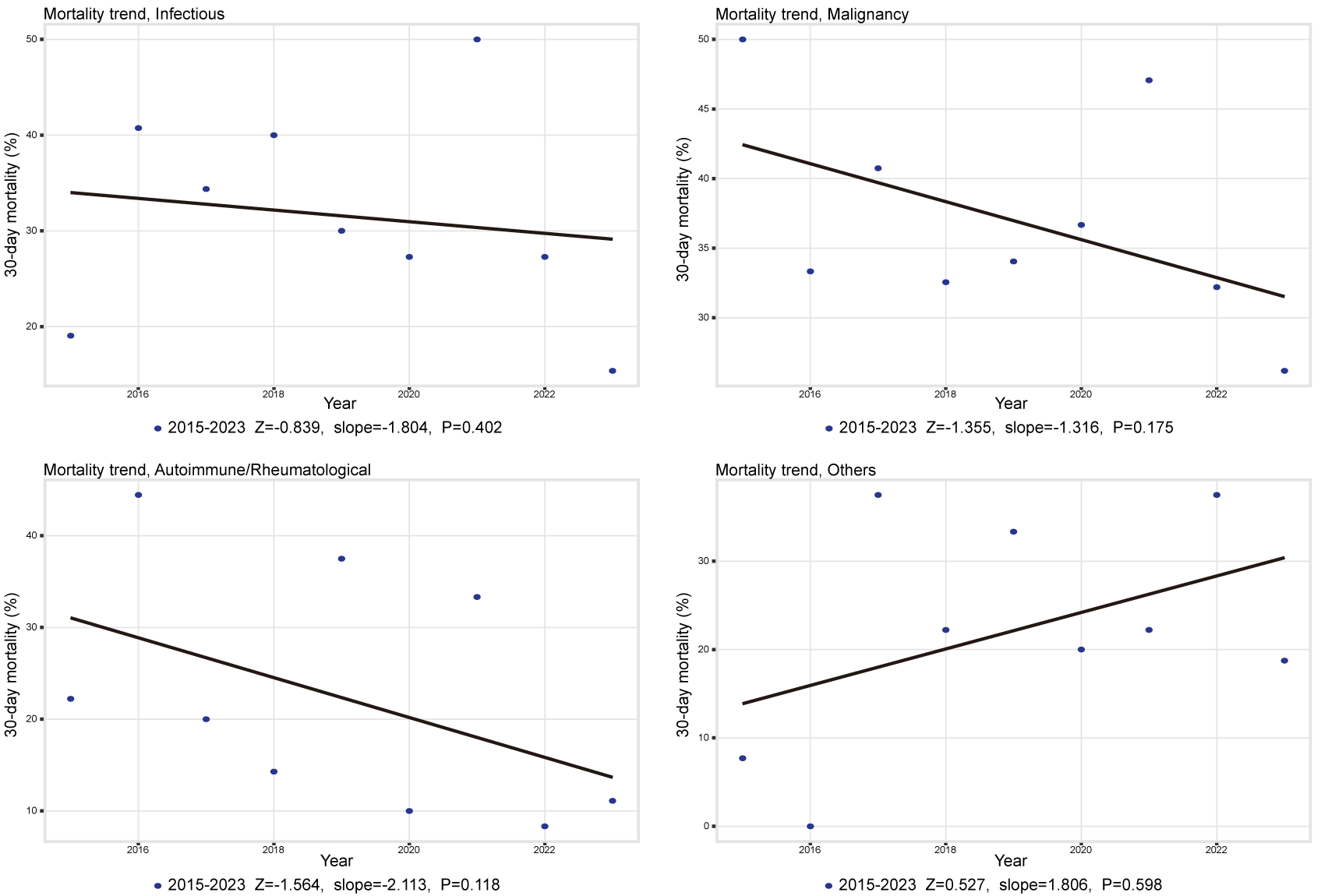


**Figure S2 30-day mortality trend for different triggers associated HLH.**


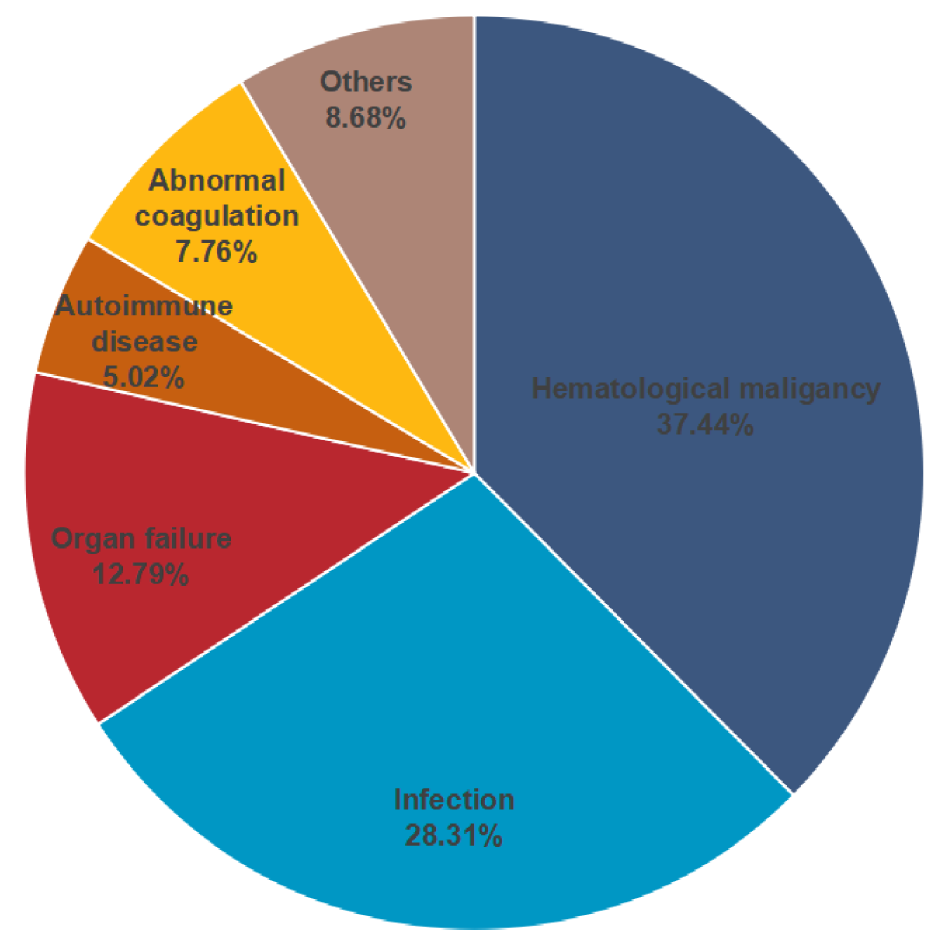


**Figure S3 Causes of adult HLH 30-day death.**


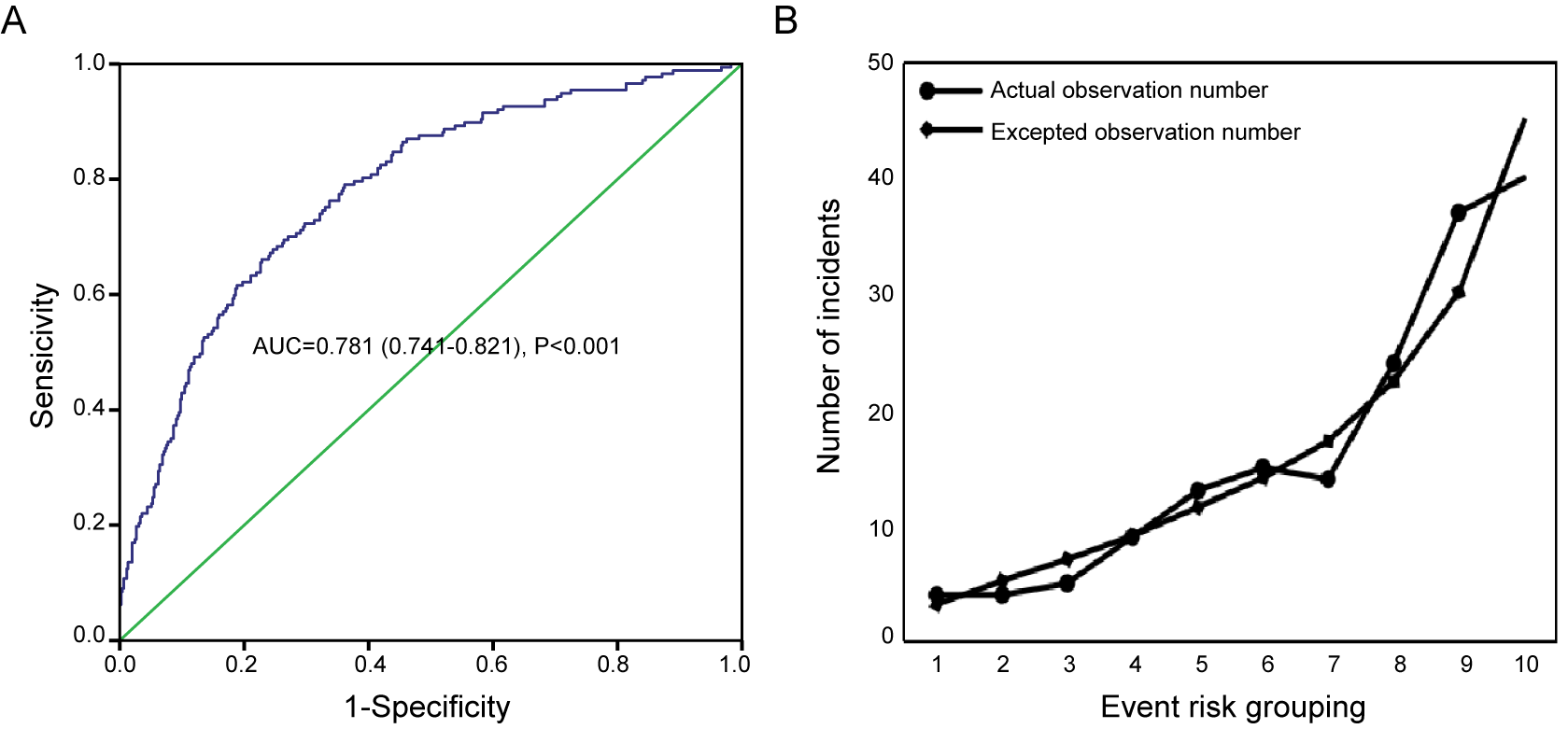


**Figure S4 Performance of the models. (A) Characteristic feature of the curve for model; (B) Calibration curves for 30-day mortality, which are indicative of predictive accuracy.**


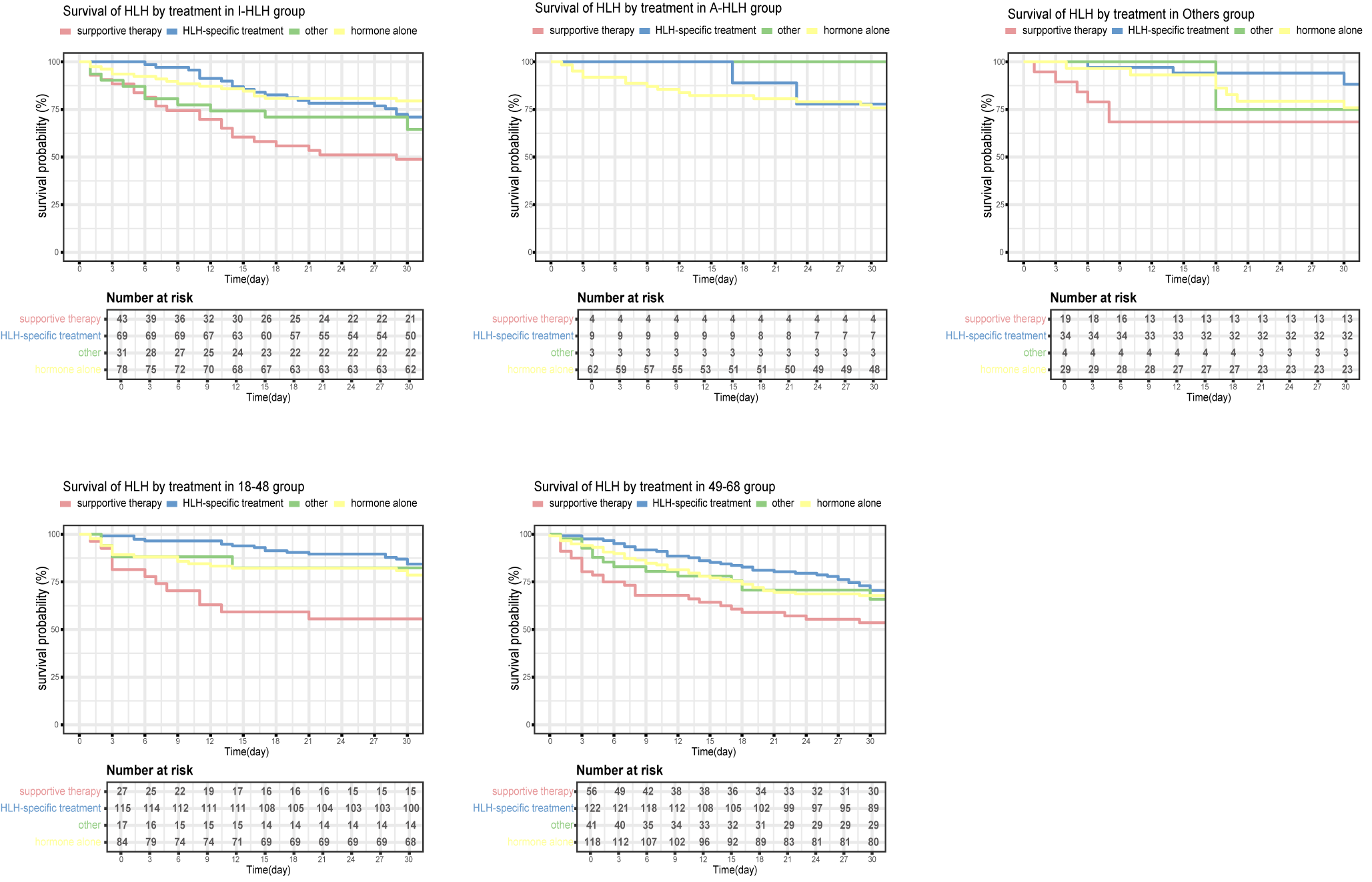


**Figure S5 30-day survival estimates by treatment.**


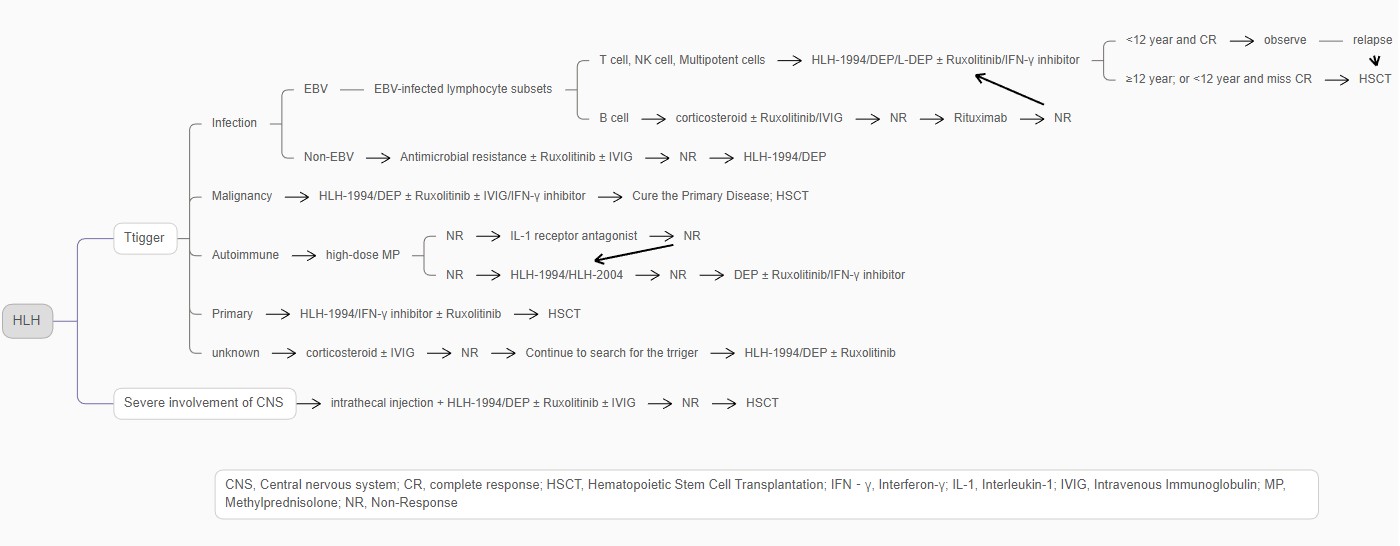


**Figure S6 The treatment protocol outlined in the Chinese guide**
